# Alternative structured spectral gradient algorithms for solving nonlinear least-squares problems

**DOI:** 10.1016/j.heliyon.2021.e07499

**Published:** 2021-07-07

**Authors:** Mahmoud Muhammad Yahaya, Poom Kumam, Aliyu Muhammed Awwal, Sani Aji

**Affiliations:** a Center of Excellence in Theoretical and Computational Science (TaCS–CoE) and KMUTTFixed Point Research Laboratory, Room SCL 802 Fixed Point Laboratory Science Laboratory Building, Department of Mathematics, Faculty of Science, King Mongkut's University of Technology Thonburi (KMUTT), 126 Pracha-Uthit Road, Bang Mod, Thung Khru, Bangkok 10140, Thailand; b NCAO Research Center, Fixed Point Theory and Applications Research Group, Center of Excellence in Theoretical and Computational Science (TaCS-CoE), Faculty of Science, King Mongkut's University of Technology Thonburi (KMUTT), 126 Pracha-Uthit Road, Bang Mod, Thung Khru, Bangkok 10140, Thailand; c Department of Mathematics, Faculty of Science, King Mongkut's University of Technology Thonburi, 126 Pracha-Uthit Road, Bang Mod, Thung Khru, Bangkok 10140, Thailand; d Department of Mathematics, Faculty of Science, Gombe State University, Gombe 760214, Nigeria

**Keywords:** Iterative algorithm, Spectral gradient algorithm, Nonlinear least squares, Line–search, Quasi–Newton algorithm

## Abstract

The study of efficient iterative algorithms for addressing nonlinear least-squares (NLS) problems is of great importance. The NLS problems, which belong to a special class of unconstrained optimization problems, are of particular interest because of the special structure of their gradients and Hessians. In this paper, based on the spectral parameters of Barzillai and Borwein (1998), we propose three structured spectral gradient algorithms for solving NLS problems. Each spectral parameter in the respective algorithms incorporates the structured gradient and the information gained from the structured Hessian approximation. Moreover, we develop a safeguarding technique for the first two structured spectral parameters to avoid negative curvature directions. Moreso, using a nonmonotone line-search strategy, we show that the proposed algorithms are globally convergent under some standard conditions. The comparative computational results on some standard test problems show that the proposed algorithms are efficient.

## 1 Introduction

Consider the general unconstrained optimization problem:

(1.1) $$\min\{f(x):\;x\in R^{n}\},$$

where $f:R^{n}\to \;R$ is assumed to be twice continuously differentiable function and bounded below. Popular iterative algorithms, such as Newton's algorithm and quasi-Newton algorithms, generate a sequence of iterates $\{x_{k}\}\subset R^{n}$ that eventually converges to some solutions of problem (1.1) using the following recurrence relation

(1.2) $$x_{k+1}=x_{k}+\alpha_{k}d_{k},$$

where the scalar $\alpha_{k}>0$ is a step size usually computed through suitable line-search strategies while the vector $d_{k}$ is a search direction obtained using different types of approaches. One of the famous and successful algorithms for calculating the search direction, $d_{k}$, is the quasi-Newton approach defined as follows:

(1.3) $$d_{k}=-B_{k}^{-1}g_{k},\;\;B_{k}\approx H_{k}\;\;\text{or}\;\;d_{k}=-Q_{k}g_{k},\;\;Q_{k}\approx H_{k}^{-1},$$

where $H_{k}$ and $g_{k}$ are the Hessian matrix and the gradient of *f* at $x_{k}$, respectively, and $B_{0}=I$, *I* is an identity matrix. The approximations $B_{k}$ and $Q_{k}$ are usually required to satisfy the following secant equations

(1.4) $$\left\{ \begin{array}{c} B_{k}s_{k-1}=y_{k-1},\\ Q_{k}y_{k-1}=s_{k-1}, \end{array}\right.$$

where $s_{k-1}=x_{k}-x_{k-1}$ and $y_{k-1}=g_{k}-g_{k-1}$.

Researchers developed quasi-Newton algorithms to address the high computational cost associated with computing the exact Hessian matrix $H_{k}$ in the famous Newton's algorithm, where the second derivative of $f(x_{k})$ needs to be evaluated in every iteration. However, most variants of quasi-Newton algorithms still need to form and store n×n matrices in every iteration. This also makes those algorithms computationally expensive and unsuitable to handle large-scaled problems. Therefore, algorithms that do not require the storage of any matrix should be a better alternative.

One of such matrix-free algorithms is called Barzilai and Borwein (BB) algorithm [1], this algorithm uses (1.2) to update its sequence of iterates where the search direction is given by $d_{k}=-g_{k}$. The scalar $\alpha_{k}$ is determined as follows. Let $D_{k}=\alpha_{k}I$ be the approximation of the Hessian matrix, the diagonal matrix $D_{k}$ is supposed to satisfy the quasi-Newton equation (1.4) where *I* is an identity matrix. But, it's often difficult to find an $\alpha_{k}$ such that $D_{k}^{-1}=\alpha_{k}^{-1}I$ satisfies the secant equation (1.4), when the entries of $x_{k}$ are more than one. As a result, Barzilai and Borwein required $D_{k}^{-1}$ to approximately satisfy the quasi-Newton equation (1.4) by finding $\alpha_{k}\in R$ that minimizes the following least squares problem

(1.5) $$\min_{\alpha }\frac{1}{2}{\left\|\alpha s_{k-1}-y_{k-1}\right\|}^{2}.$$

The solution of problem (1.5) is as follows

(1.6) $$\alpha_{k}^{BB1}=\frac{{\left\|s_{k-1}\right\|}^{2}}{s_{k-1}^{T}y_{k-1}}.$$

Similarly, we can have another choice of $\alpha_{k}$ by minimizing:

$$\min_{\alpha }\frac{1}{2}{\left\|s_{k-1}-\alpha^{-1}y_{k-1}\right\|}^{2},$$

where the solution of the problem is as follows

(1.7) $$\alpha_{k}^{BB2}=\frac{s_{k-1}^{T}y_{k-1}}{{\left\|y_{k-1}\right\|}^{2}}.$$

It is pertinent to point out here that for general unconstrained optimization problems, the BB algorithm with the spectral parameter (1.7) performs numerically better than the spectral parameter (1.6) for some problems (see, [2]). However, despite the simplicity and good performance of the BB algorithm, the spectral parameters may produce negative values for non-convex objective functions [2]. To overcome this, Raydan et al. [3] set a bound for the spectral parameter in the interval $\left[10^{-30},10^{30}\right]$. However, the interval $\left[10^{-30},10^{30}\right]$ looks artificial and therefore, a geometric mean of (1.6) and (1.7) was proposed and analyzed by Dai et al. [2]. This geometric mean is given by:

(1.8) $$\alpha_{k}^{⁎}=\frac{\left\|s_{k-1}\right\|}{\left\|y_{k-1}\right\|}.$$

This paper deals with the special class of problem (1.1), called “nonlinear least-squares problems.” The problem is defined as follows:

(1.9) $$\min_{x\in R^{n}}f(x),\;f(x)=\frac{1}{2}{\left\|R(x)\right\|}^{2},$$

where $R:R^{n}\to \;R^{m}$ (usually m≥n) is continuous and bounded below. $R(x)={\left(R_{1}(x),\ldots ,R_{m}(x)\right)}^{T}$, $R_{j}:R^{n}\to \;R$, j=1,2,…,m is twice differentiable function and ‖.‖ is the Euclidean norm.

The gradient, ∇f(x) and the Hessian, $\nabla^{2}f(x)$ of the objective function (1.9) have special structures which are respectively given by:

(1.10) $$\nabla f(x)=\sum_{j=1}^{m}R_{j}(x)\nabla R_{j}(x)=J{\left(x\right)}^{T}R(x),$$

(1.11) $$\nabla^{2}f(x)=\sum_{j=1}^{m}\nabla R_{j}(x)\nabla R_{j}{\left(x\right)}^{T}+\sum_{j=1}^{m}R_{j}(x)\nabla^{2}R_{j}(x)=J{\left(x\right)}^{T}J(x)+P(x),$$

respectively, where, $J(x)=R^{\prime }(x)$ is the Jacobian matrix of the residual function and $P(x)=\sum_{j=1}^{m}R_{j}(x)S_{j}(x)$, where $R_{j}(x)$ is *j*-component of the residual vector R(x) and $S_{j}(x)$ is the Hessian matrix of $R_{j}(x)$.

The algorithms for solving (1.9) include Newton-like approaches such as the Gauss-Newton algorithm (GN), Levenberg-Marquardt algorithm (LM), and structured quasi-Newton algorithm. In another vein, trust-region algorithms are another algorithms for solving (1.9). These algorithms do not use line–search, instead they generate steps using a quadratic model of the objective function. Some of the variations of these algorithms include the Quasi-Newton trust-region proposed by Sun et al. [4] and Adaptive trust-region algorithms developed by Sheng et al. [5]. For details about these algorithms, the interested reader may refer to the survey of algorithms for solving nonlinear least-squares problems by Mohammad et al. [6] and Yuan [7].

Guass-Newton and Levenberg-Marquardt algorithms are efficient algorithms for solving small-scale problems; however, these algorithms tend to show poor performance when applied to solve non-zero residual problems [8]. As a result of this shortcoming, Brown and Dennis [9] introduced the Structured Quasi-Newton algorithm (SQN), which utilizes GN's and quasi-Newton's step to exploit the structure of the Hessian of the objective function (1.9). The SQN algorithm shows remarkable improvement numerically upon comparison to the GN and LM algorithms (see, [10], [11], [12]). However, SQN algorithms need to compute and store matrices in each iteration. That limits their performance when solving large–scale problems. Consequently, structured matrix-free algorithms for solving (1.9) are more preferable [13], [14], [15]. For instance, Kobayashi et al. [16] introduced a structured matrix-free algorithm that uses conjugate gradient direction to solve large–scale nonlinear least-squares problems. Their algorithm incorporated some approaches such as GN, LM, and SQN into the Dai and Liao conjugate gradient algorithm [17]. They showed the global convergence of the algorithm under some standard assumptions.

In a different approach, Mohammad and Waziri [18] proposed two BB-like algorithms for solving nonlinear least-squares problems. The two algorithms update their search directions by incorporating a structured vector, which approximately satisfies the structured secant equation, into the BB spectral parameters (1.7) and (1.8). However, they derived their structured vector by approximating both the first term and the second term of the Hessian matrix (1.11) which we believe may lead to some loss of information of the Hessian matrix. In this paper, we propose three alternative matrix-free algorithms with a different structured vector obtained by retaining the first term of (1.11) and approximate its second term, since computing the exact second term is computationally expensive. As a result, our proposed search directions possess more information about the Hessian of the objective function. More so, to avoid negative curvature directions, we provide a safeguarding technique for the first two of the proposed spectral parameters when they are nonpositive at a particular iteration. Our modification improves the numerical performance of Mohammad and Waziri [18]. Numerical experiments in Section 4 support this claim.

We segmented the remainder of the paper into the following components. In the $2^{nd}$ section, we present the formulation of the proposed algorithms. We describe the global convergence of the proposed algorithms in the $3^{rd}$ section. In the $4^{th}$ section, we present numerical experiments. Finally, we give some conclusions in the $5^{th}$ Section.

## 2 Formulation of the three spectral algorithms and their algorithms

In this segment, we provide important components for the proposed algorithms. Consider the second term, P(x), of the Hessian matrix (1.11) at certain iteration, say, k−1, for k>0, i.e.,

(2.1) $$P(x_{k-1})=\sum_{j=1}^{m}R_{j}(x_{k-1})\nabla^{2}R_{j}(x_{k-1}).$$

We wish to derive a structured vector, say $\gamma_{k-1}$, in such a way that the matrix $P(x_{k-1})$ satisfies the following secant equation:

(2.2) $$P(x_{k})s_{k-1}=\gamma_{k-1},$$

where $s_{k-1}=x_{k}-x_{k-1}$. Using Taylor's series expansion on $\nabla R_{j}(x_{k-1})$ and simplifying it, will give

(2.3) $$\nabla R_{j}(x_{k-1})\approx \nabla R_{j}(x_{k})-\nabla^{2}R_{j}{\left(x_{k}\right)}^{T}s_{k-1},\;\;j=1,2,3,\ldots ,m,$$

therefore, pre-multiplying (2.3) by $R_{j}(x_{k})$ and simplifying gives

(2.4) $$R_{j}(x_{k})\nabla^{2}R_{j}{\left(x_{k}\right)}^{T}s_{k-1}\approx R_{j}(x_{k})\nabla R_{j}(x_{k})-R_{j}(x_{k})\nabla R_{j}(x_{k-1}).$$

Adding up on both sides of the above equation (2.4) for j=1,2,3,…,m and using (2.1), it gives

(2.5) $$P(x_{k})s_{k-1}\approx {\left(J(x_{k})-J(x_{k-1})\right)}^{T}R(x_{k}).$$

Multiplying equation (1.11) by $s_{k-1}$ and substituting (2.5) into it, we have

(2.6) $$\nabla^{2}f(x_{k})s_{k-1}\approx J{\left(x_{k}\right)}^{T}J(x_{k})s_{k-1}+{\left(J(x_{k})-J(x_{k-1})\right)}^{T}R(x_{k}).$$

Suppose $D_{k}\approx \nabla^{2}f(x_{k})$, such that

(2.7) $$D_{k}s_{k-1}\approx \gamma_{k-1},$$

is satisfied. Then from (2.6), we have

(2.8) $$\gamma_{k-1}=J{\left(x_{k}\right)}^{T}J(x_{k})s_{k-1}+{\left(J(x_{k})-J(x_{k-1})\right)}^{T}R(x_{k}).$$

In a similar manner to classical *BB* method, suppose $D_{k}=\alpha_{k}I$, we require $D_{k}^{-1}$ to approximately satisfy the above modified secant equation (2.7) by finding $\alpha_{k}\in R$ that minimize the following least–squares problems

(2.9) $$\min_{\alpha }\frac{1}{2}{\left\|\alpha s_{k-1}-\gamma_{k-1}\right\|}^{2},$$

the solution of problem (2.9) is as follows

(2.10) $$\alpha_{k}^{M1}=\frac{{\left\|s_{k-1}\right\|}^{2}}{s_{k-1}^{T}\gamma_{k-1}}.$$

Similarly, another choice of $\alpha_{k}$ obtained by minimizing:

$$\min_{\alpha }\frac{1}{2}{\left\|s_{k-1}-\alpha^{-1}\gamma_{k-1}\right\|}^{2},$$

the above minimization problem has the following solution given as

(2.11) $$\alpha_{k}^{M2}=\frac{s_{k-1}^{T}\gamma_{k-1}}{{\left\|\gamma_{k-1}\right\|}^{2}}.$$

The geometric mean of $\alpha_{k}^{BB1}$ and $\alpha_{k}^{BB2}$ parameters is given by:

(2.12) $$\alpha_{k}^{M3}=\frac{\left\|s_{k-1}\right\|}{\left\|\gamma_{k-1}\right\|}.$$

To build the proposed algorithms, we define the search directions using the above spectral parameters in equations (2.10), (2.11) and (2.12) as follows

(2.13) $$\left\{ \begin{array}{c} d_{k}^{M1}=-\alpha_{k}^{M1}g_{k},\\ d_{k}^{M2}=-\alpha_{k}^{M2}g_{k},\\ d_{k}^{M3}=-\alpha_{k}^{M3}g_{k}, \end{array}\right.$$

where

(2.14) $$\left\{ \begin{array}{c} \alpha_{k}^{M1}=\frac{{\left\|s_{k-1}\right\|}^{2}}{s_{k-1}^{T}\gamma_{k-1},}\\ \alpha_{k}^{M2}=\frac{s_{k-1}^{T}\gamma_{k-1}}{{\left\|\gamma_{k-1}\right\|}^{2}},\\ \alpha_{k}^{M3}=\frac{\left\|s_{k-1}\right\|}{\left\|\gamma_{k-1}\right\|}. \end{array}\right.$$

In the case of the search directions $d_{k}^{M1}$ and $d_{k}^{M2}$, negative curvature directions could be avoided by making $s_{k-1}^{T}\gamma_{k-1}$ strictly positive. As a result, a suitable safeguarding parameter which is updated in every iteration is usually used to replace $s_{k-1}^{T}\gamma_{k-1}$ i.e. when $s_{k-1}^{T}\gamma_{k-1}\leq 0$. For example, Luengo et al. [19] developed a retarding technique, that is, if $s_{k-1}^{T}\gamma_{k-1}\leq 0$ then $\alpha_{k}=\vartheta \alpha_{k-1}$, where *ϑ* is a suitable positive constant. Recently, Mohammad and Waziri [20] presented another safeguarding parameter $\eta_{k}=\max\{\vartheta \alpha_{k-1},s_{k-1}^{T}\gamma_{k-1}+\|s_{k-1}\|\|\gamma_{k-1}\|\}$ to replace $s_{k-1}^{T}\gamma_{k-1}$ whenever it is nonpositive.

Similarly, we propose another safeguarding technique for the spectral parameters in order to avoid a negative curvature direction. Obviously, $\alpha_{k}^{M1}$ and $\alpha_{k}^{M2}$ will be nonpositive only if $s_{k-1}^{T}\gamma_{k-1}\leq 0$. Therefore, we present the safeguarding technique as follows:

Since it holds that $s_{k-1}^{T}\gamma_{k-1}\leq 0.5({\left\|s_{k-1}\right\|}^{2}+{\left\|\gamma_{k-1}\right\|}^{2})$, then if $s_{k-1}^{T}\gamma_{k-1}\leq 0$ in $\alpha_{k}^{M1}$ or $\alpha_{k}^{M2}$, we replace it with the following parameter

(2.15) $$\eta_{k}^{⁎}=\max\{\vartheta \alpha_{k-1},\;{\left\|s_{k-1}\right\|}^{2}+{\left\|\gamma_{k-1}\right\|}^{2}\},$$

where *ϑ* is a positive constant. In the case of $\alpha_{k}^{M3}$, it can be seen that computing $s_{k-1}^{T}\gamma_{k-1}$ is not required, and as a result, $\alpha_{k}^{M3}$, is expected to perform reasonably well. The preliminary numerical experiments we conducted support our expectations.

To ensure the global convergence of the proposed algorithms, we adopt the nonmonotone line-search of Zhang and Hager [21]. We describe the nonmonotone line-search as follows. Suppose that a direction $d_{k}$ (that is, either $d_{k}^{M1}$, $d_{k}^{M2}$ or $d_{k}^{M3}$) is sufficiently descent, then a step length *h* is determined such that it satisfies the following nonmonotone Armijo-type line-search conditions:

(2.16) $$f(x_{k}+hd_{k})\leq U_{k}+\delta hg_{k}^{T}d_{k},\;\delta \in (0,1),\;g_{k}=J_{k}^{T}R_{k},$$

where,

(2.17) $$\left\{ \begin{array}{c} U_{0}=f(x_{0}),\\ U_{k+1}=\frac{\mu_{k}W_{k}U_{k}+f(x_{k+1})}{W_{k+1}},\\ W_{0}=1,\\ W_{k+1}=\mu_{k}W_{k}+1, \end{array}\right.$$

and $J_{k}=J(x_{k})$, $R_{k}=R(x_{k})$.

The degree of nonmonotonicity is controlled by $\mu_{k}$. If for all $k,\;\mu_{k}=0$, then the nonmonotone line-search (2.16) above usually reduces to the Wolfe or Armijo-type line-search.

Remark 2.1 [21] The sequence $\left\{U_{k}\right\}$ lies between $f(x_{k})$ and $C_{k}$, where

(2.18) $$C_{k}:=\frac{1}{k+1}\sum_{j=0}^{k}f(x_{j}),\;\forall k\geq 0.$$

Next, we outline the steps of the proposed alternative structured spectral algorithms for solving nonlinear least-squares problems.

**Algorithm 1:**
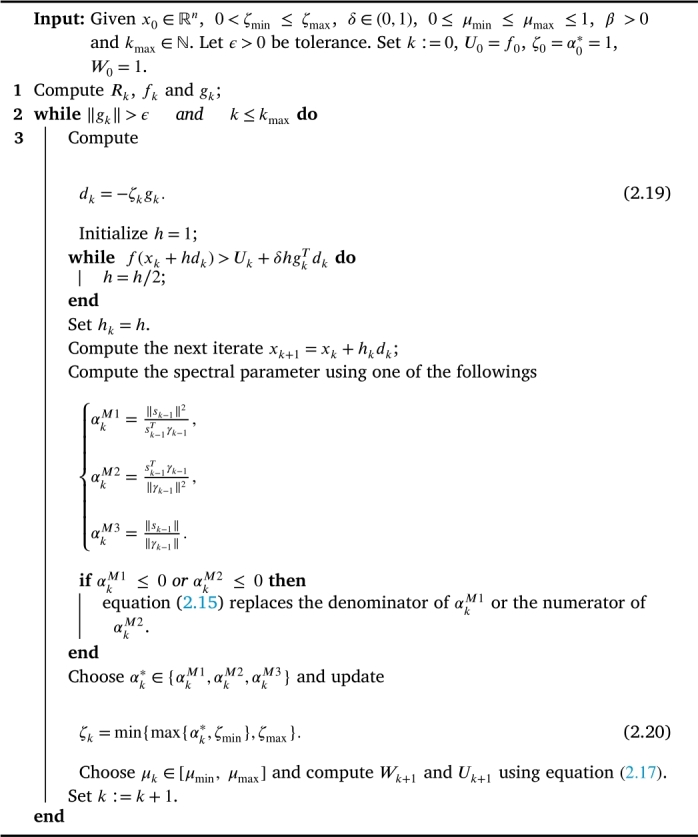
Alternative structured spectral algorithm (ASSA).

Remark 2.2 It is important to point out that, the above algorithm is composed of three different algorithms combined in one, different choices of the parameters $\alpha_{k}^{M1}$, $\alpha_{k}^{M2}$ and $\alpha_{k}^{M3}$ correspond to different algorithms. If $\alpha_{k}^{M1}$ or $\alpha_{k}^{M2}$ or $\alpha_{k}^{M3}$ is used, we denoted the algorithm as **ASSA1** or **ASSA2** or **ASSA3**, respectively. Also, for each problem considered, we write MATLAB code for the structured gradient $g_{k}$ and the structured vector $\gamma_{k-1}$, in such a way that the matrix-vector product components of the vectors are computed directly without explicitly forming or storing a matrix throughout the iteration process.

## 3 Convergence analysis

To discuss the global convergence of the proposed algorithms, we first state some valuable assumptions as follows:

Assumption 3.1 The level set $\ell =\{x\in R^{n}|f(x)\leq f(x_{o})\}$ is bounded, i.e. there exists a positive constant *ν* such that ‖x‖≤ν, ∀x∈ℓ.

Assumption 3.2 The Jacobian matrix $J(x)=\nabla R{\left(x\right)}^{T}$ is Lipschitz continuous on some neighborhood *N* of *ℓ* with Lipschitz constant $p_{1}$ i.e. $\left\|J(x)-J(y)\|\leq p_{1}\|x-y\right\|$, ∀x,y∈ℓ.

It can be deduced from the above Assumption 3.2 that there exist positive constants $p_{2},p_{3},q_{1},q_{2},q_{3}$ such that for every x,y∈ℓ, for which

$$\|R(x)-R(y)\|\leq p_{2}\|x-y\|,\|g(x)-g(y)\|\leq p_{3}\|x-y\|,\|J(x)\|<q_{1},\;\;\;\|R(x)\|<q_{2},\;\;\;\|g(x)\|\leq q_{3}.$$

Lemma 3.3 *Suppose* Assumption 3.1, Assumption 3.2 *hold, then there exists a positive constant L such that,* ∀k>0*,*

(3.1) $$\|\gamma_{k-1}\|\leq L\|s_{k-1}\|.$$

Proof $$\|\gamma_{k-1}\|=\|J{\left(x_{k}\right)}^{T}J(x_{k})s_{k-1}+{\left(J(x_{k})-J(x_{k-1})\right)}^{T}R(x_{k})\|\leq \|J{\left(x_{k}\right)}^{T}J(x_{k})s_{k-1}\|+\|{\left(J(x_{k})-J(x_{k-1})\right)}^{T}R(x_{k})\|\;\;(\text{using triangular inequality})\leq {\left\|J(x_{k})\right\|}^{2}\|s_{k-1}\|+\|J(x_{k})-J(x_{k-1})\|\|R(x_{k})\|\;\;(\text{using matrix norm property })\leq q_{1}^{2}\|s_{k-1}\|+p_{1}\|x_{k}-x_{k-1}\|\|R(x_{k})\|\leq q_{1}^{2}\|s_{k-1}\|+p_{1}q_{2}\|s_{k-1}\|=(q_{1}^{2}+p_{1}q_{2})\|s_{k-1}\|.$$

Hence, by setting $L:=q_{1}^{2}+p_{1}q_{2}$, the inequality (3.1) holds. □

Lemma 3.4 *The parameter* $\zeta_{k}$ *defined by* (2.20) *is well-defined and bounded for every* k≥0*.*

Proof For k=0, $\zeta_{0}=\alpha_{0}^{⁎}=1$.

Now, for k≥1, we consider the following three choices for $\alpha_{k}^{⁎}$.

**Choice 1:** $\alpha_{k}^{⁎}=\alpha_{k}^{M1}=\frac{{\left\|s_{k-1}\right\|}^{2}}{s_{k-1}^{T}\gamma_{k-1}}$. Clearly, if $s_{k-1}^{T}\gamma_{k-1}$ is positive, then $\alpha_{k}^{⁎}>0$ and as a result $\zeta_{k}>0$. Else, set $\alpha_{k}^{⁎}=\frac{{\left\|s_{k-1}\right\|}^{2}}{\max\{\vartheta \alpha_{k-1},\;{\left\|s_{k-1}\right\|}^{2}+{\left\|\gamma_{k-1}\right\|}^{2}\}}$. If $\vartheta \alpha_{k-1}>{\left\|s_{k-1}\right\|}^{2}+{\left\|\gamma_{k-1}\right\|}^{2}$ then, it is obvious that $\alpha_{k}^{⁎}>0$ which means $\zeta_{k}>0$. Otherwise, if $({\left\|s_{k-1}\right\|}^{2}+{\left\|\gamma_{k-1}\right\|}^{2})>\vartheta \alpha_{k-1}$, then we have

$$\alpha_{k}^{⁎}=\frac{{\left\|s_{k-1}\right\|}^{2}}{{\left\|s_{k-1}\right\|}^{2}+{\left\|\gamma_{k-1}\right\|}^{2}}\geq \frac{{\left\|s_{k-1}\right\|}^{2}}{{\left\|s_{k-1}\right\|}^{2}+L^{2}{\left\|s_{k-1}\right\|}^{2}}=\frac{{\left\|s_{k-1}\right\|}^{2}}{(L^{2}+1){\left\|s_{k-1}\right\|}^{2}}=\frac{1}{L^{2}+1}>0,$$

where the inequality follows from (3.1).

**Choice 2:** $\alpha_{k}^{⁎}=\alpha_{k}^{M2}=\frac{s_{k-1}^{T}\gamma_{k-1}}{{\left\|s_{k-1}\right\|}^{2}}$.

If $s_{k-1}^{T}\gamma_{k-1}>0$, then $\alpha_{k}^{⁎}>0$ and subsequently $\zeta_{k}>0$. Else, we set $\alpha_{k}^{⁎}=\frac{\max\{\vartheta \alpha_{k-1},\;{\left\|s_{k-1}\right\|}^{2}+{\left\|\gamma_{k-1}\right\|}^{2}\}}{{\left\|s_{k-1}\right\|}^{2}}$.

If $\vartheta \alpha_{k-1}>{\left\|s_{k-1}\right\|}^{2}+{\left\|\gamma_{k-1}\right\|}^{2}$, then $\alpha_{k}^{⁎}>0$ which means $\zeta_{k}>0$.

Otherwise, if ${\left\|s_{k-1}\right\|}^{2}+{\left\|\gamma_{k-1}\right\|}^{2}>\vartheta \alpha_{k-1}$, then

$$\alpha_{k}^{⁎}=\frac{{\left\|s_{k-1}\right\|}^{2}+{\left\|\gamma_{k-1}\right\|}^{2}}{{\left\|\gamma_{k-1}\right\|}^{2}}\geq \frac{\frac{1}{L^{2}}{\left\|\gamma_{k-1}\right\|}^{2}+{\left\|\gamma_{k-1}\right\|}^{2}}{{\left\|\gamma_{k-1}\right\|}^{2}}=\frac{\left(\frac{1}{L^{2}}+1\right)}{{\left\|\gamma_{k-1}\right\|}^{2}}{\left\|\gamma_{k-1}\right\|}^{2}=\frac{1}{L^{2}}+1>0,$$

where the inequality follows from (3.1).

**Choice 3:**

$$\alpha_{k}^{⁎}=\alpha_{k}^{M3}=\frac{\left\|s_{k-1}\right\|}{\left\|\gamma_{k-1}\right\|}\geq \frac{\left\|s_{k-1}\right\|}{L\|s_{k-1}\|}=\frac{1}{L}>0,$$

where the inequality follows from (3.1). Therefore, from choices 1, 2, and 3 above, it follows that $\alpha_{k}^{⁎}$ is well-defined for every three choices. Thus, $\zeta_{k}$ is well-defined. Also, from the definition of $\zeta_{k}$ in (2.20), it follows that for all k≥0, $\zeta_{k}$ is bounded above and below by $\zeta_{\max}$ and $\zeta_{\min}$ respectively.

Hence, combining all the cases, we have

$$\max\{\frac{1}{L^{2}+1},\zeta_{\min}\}\leq \zeta_{k}\leq \zeta_{\max}.$$

 □

Lemma 3.5 *Suppose the sequence* $\left\{x_{k}\right\}$ *and the search direction* $\left\{d_{k}\right\}$ *are generated by* Algorithm 1*. Let* $c_{1}$ *and* $c_{2}$ *be two positive constants. Then for all* k≥0*, the following inequalities hold:*

(a) $g_{k}^{T}d_{k}\leq -c_{1}{\left\|g_{k}\right\|}^{2}$*,*
(b) $\left\|d_{k}\|\leq c_{2}\|g_{k}\right\|$*,*
(c) $f_{k}\leq U_{k}\leq C_{k}$*.*

Proof For inequality (*a*), suppose $d_{k}$ is defined by (2.19), then

(3.2) $$g_{k}^{T}d_{k}=-\zeta_{k}{\left\|g_{k}\right\|}^{2}\leq -\max\{\frac{1}{L^{2}+1},\;\zeta_{\max}\}{\left\|g_{k}\right\|}^{2},$$

the result follows by setting $c_{1}=\max\{\frac{1}{L^{2}+1},\;\zeta_{\max}\}$. The inequality in (*b*) follows from Lemma 3.4. To show the inequality (*c*), let t≥0 and define Φ:R→R by

(3.3) $$\Phi =\frac{tU_{k-1}+f(x_{k})}{t+1}.$$

Differentiating the above (3.3) with respect to *t* gives

(3.4) $$\frac{d\Phi }{dt}=\frac{U_{k-1}-f(x_{k})}{{\left(t+1\right)}^{2}}.$$

By the relation $g_{k}^{T}d_{k}\leq -c_{1}{\left\|g_{k}\right\|}^{2},\;\forall \;k$, we have from (2.16) that

$$f(x_{k})=f(x_{k-1}+hd_{k-1})\leq U_{k-1}+\delta hg_{k-1}^{T}d_{k-1}\leq U_{k-1}-c_{1}\delta h{\left\|g_{k-1}\right\|}^{2}\leq U_{k-1}.$$

This means, for all t≥0, $\frac{d\Phi }{dt}\geq 0$ which thus implies that Φ is nondecreasing. Hence (3.3) satisfies $f(x_{k})=\Phi (0)\leq \Phi (t)$, for all t≥0. In particular, by taking $t=\mu_{k-1}W_{k-1}$ there follows

(3.5) $$f(x_{k})=\Phi (0)\leq \Phi (\mu_{k-1}W_{k-1})=\frac{\mu_{k-1}W_{k-1}U_{k-1}+f(x_{k})}{\mu_{k-1}W_{k-1}+1}=\frac{\mu_{k-1}W_{k-1}U_{k-1}+f(x_{k})}{W_{k}}\;\;(\text{from (2.17)})=U_{k}.$$

Hence, the lower bound of $U_{k}$ is established.

Next, we show $U_{k}\leq C_{k}$ by induction. Let k=0, from (2.17) we have $U_{0}=C_{0}=f(x_{0})$. Now, suppose that $U_{j}\leq C_{j}$, for all 0≤j<k. Since $\mu_{k}\in [0,1]$ and $W_{0}=1$ by (2.17) we have

(3.6) $$W_{j+1}=1+\sum_{i=0}^{j}\prod_{l=0}^{i}\mu_{j-l}\leq j+2.$$

Combining (3.3), (3.5) and (3.6), we have,

$$U_{k}=\Phi (W_{k}-1)=\Phi (\mu_{k-1}W_{k-1})=\Phi \left(\sum_{i=0}^{k-1}\prod_{n=0}^{i}\mu_{k-n-1}\right)\leq \Phi (k).$$

By induction step, we obtain

(3.7) $$\Phi (k)=\frac{kU_{k-1}+f(x_{k})}{k+1}\leq \frac{kC_{k-1}+f(x_{k})}{k+1}=C_{k}.$$

Hence it holds that $U_{k}\leq C_{k}$. □

Theorem 3.6 *Assume* f(x) *is given by* (1.9) *and* Assumption 3.1, Assumption 3.2 *hold. Then the generated sequence of iterates* $\left\{x_{k}\right\}$ *from the* Algorithm 1 *is contained in the level set ℓ and*

(3.8) $$\underset{k\to \infty }{\lim\;\inf}\|g_{k}\|=0.$$

*Furthermore, if* $\eta_{\max}<1$*, then*

(3.9) $$\lim_{k\to \infty }\|g_{k}\|=0.$$

Proof The proof follows from [21]. □

## 4 Numerical experiments

Numerical experiments are reported in this section in order to evaluate the computational performance of the proposed algorithms ASSA1, ASSA2 and ASSA3 in comparison with the algorithms (SSGM1 and SSGM2) as reported in [20].

In the experiment. Among the 35 benchmark test problems, which are considered in the experiment, 27 problems are large-scaled while the remaining 8 are small-scaled. The list of the test problems, their initial guesses, and their respective references are reported in Tables 1 and 2.

**Table 1:** List of *large–scale* test problems with their respective references and starting points.

Problem	Function name	Starting point
**P1**	Penalty function I [22]	(1/3,1/3,...,1/3)^*T*^
**P2**	Trigonometric function [23]	(1/*n*,...,1/*n*)^*T*^
**P3**	Discrete boundary-value function [23]	${\left(\frac{1}{n+1}(\frac{1}{n+1}-1),...,\frac{1}{n+1}(\frac{n}{n+1}-1)\right)}^{T}$
**P4**	Linear full rank function [23]	(1,1,...,1)^*T*^
**P5**	Linear rank-1 function [23]	(1,1,...,1)^*T*^
**P6**	Problem 202 [24]	(2,2,...2)^*T*^
**P7**	Problem 206 [24]	(1/*n*,...,1/*n*)^*T*^
**P8**	Problem 212 [24]	(0.5,...,0.5)^*T*^
**P9**	Ascher and Russel boundary value problem [24]	(1/*n*,1/*n*,...,1/*n*)^*T*^
**P10**	Strictly convex function (Raydan 1) [22]	(1/*n*,2/*n*,...,1)^*T*^
**P11**	Singular function 2 [22]	(1,1,...,1)
**P12**	Exponential function 1 [22]	(*n*/*n*−1,*n*/*n*−1,...,*n*/*n*−1)^*T*^
**P13**	Exponential function 2 [22]	${\left(1/n^{2},1/n^{2},...,1/n^{2}\right)}^{T}$
**P14**	Logarithm function [22]	(1,1,...,1)^*T*^
**P15**	Trigonometric exponential function [24]	(0.5,0.5,...,0.5)^*T*^
**P16**	Extended Powell singular function [22]	(1.5*E*−4,...,1.5*E*−4)^*T*^
**P17**	Function 21 [22]	(1,1,...,1)^*T*^
**P18**	Tridiagonal Broyden function [23]	(−1,−1,...,−1)^*T*^
**P19**	Extended Himmelblau function [25]	(1,1/*n*,1,1/*n*,...,1,1/*n*)^*T*^
**P20**	Function 27 [22]	${\left(100,1/n^{2},...,1/n^{2}\right)}^{T}$
**P21**	Trigonometric Logarithmic function [20]	(1,1,...,1)^*T*^
**P22**	Zero Jacobian function [22]	$for\;i=1,\;\frac{100(n-100)}{n},\;\;for\;i\geq 2,\;\frac{\left(n-1000)(n-500\right)}{{\left(60n\right)}^{2}}$
**P23**	Exponential function [22]	(1/(4*n*^2^),2/(4*n*^2^),...,*n*/(4*n*^2^))
**P24**	Singular Broyden function 1 [24]	(−1,−1,...,−1)^*T*^
**P25**	Brown almost linear function [23]	(0.5,0.5,...,0.5)^*T*^
**P26**	Extended Freudenstein and Roth function [22]	(6,3,6,3,...,6,3)^*T*^
**P27**	Generalized Tridiagonal Broyden [24]	(−1,−1,...,−1)^*T*^

**Table 2:** List of *small-scale* test problems with their respective references and starting points.

Problem	Function name	Starting point
**P28**	Brown Badly Scaled function [23]	(1,1)^*T*^
**P29**	Jennrich and Sampson function [23]	(0.2,0.2)^*T*^
**P30**	Box three-dimensional function [23]	(0,0.1)^*T*^
**P31**	Rank deficient Jacobian [26]	(−1,1)^*T*^
**P32**	Rosenbrock function [23]	(−1,1)^*T*^
**P33**	Parameterized function [27]	(10,10)^*T*^
**P34**	Freudenstein and Roth function [23]	(0.5,−2)^*T*^
**P35**	Beale Function [23]	(2,3)

For the large-scaled test problems, we solved each test problem using the following dimensions 1000, 3000, 5000, 7000, 9000, 11000, and 13000. In total, we solved 197 instances in the course of the experiments.

The parameters used in the execution of all our algorithms and SSGM algorithms are as follows:

• **ASSA Algorithms:** $\zeta_{\min}=10^{-30}$, $\zeta_{\max}=10^{30}$, $\delta =10^{-4}$, $\mu_{\min}=0.1$, $\mu_{\max}=0.85$, $\text{tolerance,}\;\epsilon =10^{-4}$ and ϑ=1000.
• **SSGM Algorithms:** All the parameters used in these algorithms remain the same as in [20].

The coding of all the algorithms and their execution were done in MATLAB R2019b on ACER-PC with intel (8th generation) CORE i5-8265U @ CPU 1.60 GHz processor and 8 GB of RAM. The iteration process proceeds as long as the inequality $\|g_{k}\|>\epsilon =10^{-4}$ holds. Termination of the iteration will occur when the above inequality is FALSE, or the number of iterations exceeds 1000, or the function evaluation surpasses 5000. In all the above-stated instances, success is reported only if the inequality $\|g_{k}\|\leq \epsilon =10^{-4}$ is satisfied otherwise, a failure represented by *F*, is reported.

The results of the experiments as reported in Tables 3, 4, 5 and 6 show the number of iterations *(ITER)*, the number of function evaluations *(FVAL)*, and the time taken required by an algorithm to approximately converge to a solution *(CPU-TIME)*.

**Table 3:** Numerical results of ASSA algorithms and SSGM algorithms on large-scaled problems 1 - 9 with their dimensions.

PROBLEMS	DIM	ASSA1			SSGM1			ASSA2			SSGM2			ASSA3		
		ITER	FEVAL	CPU–TIME	ITER	FEVAL	CPU–TIME	ITER	FEVAL	CPU–TIME	ITER	FEVAL	CPU–TIME	ITER	FEVAL	CPU–TIME
**P1**	**1000**	4	5	0.1218	4	5	0.0040	4	5	0.0525	4	5	0.0139	4	5	0.0314
	**3000**	3	4	0.0475	4	5	0.0087	3	4	0.0275	4	5	0.0160	3	4	0.0332
	**5000**	3	4	0.0194	3	4	0.0181	3	4	0.0212	3	4	0.0155	3	4	0.0110
	**7000**	3	4	0.0170	3	4	0.0296	3	4	0.0308	3	4	0.0162	3	4	0.0399
	**9000**	3	4	0.0198	3	4	0.0174	3	4	0.0333	3	4	0.0230	3	4	0.0722
	**11000**	2	3	0.0202	2	3	0.0130	2	3	0.0184	2	3	0.0379	2	3	0.0405
	**13000**	2	3	0.0218	2	3	0.0345	2	3	0.0341	2	3	0.0315	2	3	0.0242

**P2**	**1000**	20	40	0.1442	3	4	0.0068	20	40	0.1150	3	4	0.0144	20	40	0.0527
	**3000**	18	42	0.0842	F	F	F	20	44	0.1984	F	F	F	19	43	0.2013
	**5000**	23	48	0.2431	F	F	F	23	48	0.1957	F	F	F	23	48	0.2336
	**7000**	23	49	0.1707	F	F	F	24	50	0.2819	F	F	F	23	49	0.2680
	**9000**	21	48	0.2654	F	F	F	23	50	0.3223	F	F	F	22	49	0.5296
	**11000**	24	51	0.3632	F	F	F	25	52	0.4220	F	F	F	25	52	0.5226
	**13000**	22	50	0.2699	F	F	F	24	52	0.2889	F	F	F	23	51	0.6974

**P3**	**1000**	10	14	0.1322	10	14	0.0376	8	12	0.0178	8	12	0.0546	10	14	0.0522
	**3000**	4	8	0.0364	4	8	0.0283	4	8	0.0321	4	8	0.0236	3	7	0.0374
	**5000**	3	7	0.0155	3	7	0.0274	2	6	0.0478	2	6	0.0348	3	7	0.0567
	**7000**	2	6	0.0300	2	6	0.0327	2	6	0.0220	2	6	0.0285	2	6	0.0293
	**9000**	1	5	0.0345	1	5	0.0535	1	5	0.0697	1	5	0.0381	1	5	0.0554
	**11000**	1	5	0.0551	1	5	0.0308	1	5	0.0185	1	5	0.0643	1	5	0.0305
	**13000**	1	5	0.0307	1	5	0.0377	1	5	0.0274	1	5	0.0474	1	5	0.0729

**P4**	**1000**	2	3	0.0455	2	3	0.0051	2	3	0.0056	2	3	0.0045	2	3	0.0049
	**3000**	2	3	0.0098	2	3	0.0072	2	3	0.0052	2	3	0.0102	2	3	0.0057
	**5000**	2	3	0.0070	2	3	0.0258	2	3	0.0054	2	3	0.0105	2	3	0.0136
	**7000**	2	3	0.0053	2	3	0.0098	2	3	0.0204	2	3	0.0115	2	3	0.0294
	**9000**	2	3	0.0220	2	3	0.0103	2	3	0.0151	2	3	0.0170	2	3	0.0283
	**11000**	2	3	0.0110	2	3	0.0267	2	3	0.0277	2	3	0.0147	2	3	0.0524
	**13000**	2	3	0.0126	2	3	0.0178	2	3	0.0165	2	3	0.0350	2	3	0.0160

**P5**	**1000**	2	59	0.0660	F	F	F	2	59	0.0144	F	F	F	2	59	0.0235
	**3000**	3	70	0.0286	F	F	F	3	70	0.0348	F	F	F	3	70	0.0508
	**5000**	3	74	0.0956	F	F	F	3	74	0.0785	F	F	F	3	74	0.0361
	**7000**	3	77	0.0523	F	F	F	3	77	0.0445	F	F	F	3	77	0.1397
	**9000**	3	79	0.0724	F	F	F	3	79	0.1199	F	F	F	3	79	0.1095
	**11000**	3	81	0.1140	F	F	F	3	81	0.1731	F	F	F	3	81	0.1185

**P6**	**1000**	5	6	0.0569	5	6	0.0027	5	6	0.0038	5	6	0.0142	5	6	0.0047
	**3000**	5	6	0.0059	5	6	0.0086	5	6	0.0136	5	6	0.0083	5	6	0.0101
	**5000**	5	6	0.0130	5	6	0.0135	5	6	0.0202	5	6	0.0171	5	6	0.0278
	**7000**	5	6	0.0100	5	6	0.0328	5	6	0.0426	5	6	0.0295	5	6	0.0465
	**9000**	5	6	0.0307	5	6	0.0376	5	6	0.0288	5	6	0.0382	5	6	0.0765
	**11000**	5	6	0.0393	5	6	0.0360	5	6	0.0378	5	6	0.0936	5	6	0.0371
	**13000**	5	6	0.0703	5	6	0.0478	5	6	0.0416	5	6	0.0671	5	6	0.0916

**P7**	**1000**	10	14	0.1364	10	14	0.0129	8	12	0.0199	8	12	0.0186	10	14	0.0194
	**3000**	4	8	0.0176	4	8	0.0226	4	8	0.0408	4	8	0.0181	3	7	0.0103
	**5000**	3	7	0.0256	3	7	0.0530	2	6	0.0143	2	6	0.0240	3	7	0.0214
	**7000**	2	6	0.0208	2	6	0.0194	2	6	0.0432	2	6	0.0199	2	6	0.0426
	**9000**	1	5	0.0401	1	5	0.0274	1	5	0.0216	1	5	0.0498	1	5	0.0226
	**11000**	1	5	0.0167	1	5	0.0189	1	5	0.0185	1	5	0.0241	1	5	0.0656
	**13000**	1	5	0.0141	1	5	0.0215	1	5	0.0469	1	5	0.0323	1	5	0.0597

**P8**	**1000**	5	7	0.0857	6	8	0.0148	5	7	0.0097	5	7	0.0118	5	7	0.0252
	**3000**	5	7	0.0180	6	8	0.0154	5	7	0.0210	5	7	0.0253	5	7	0.0345
	**5000**	5	7	0.0171	6	8	0.0293	5	7	0.0264	6	8	0.0279	5	7	0.0582
	**7000**	5	7	0.0348	6	8	0.0531	5	7	0.0482	6	8	0.1154	5	7	0.0302
	**9000**	5	7	0.0476	6	8	0.0770	5	7	0.0772	6	8	0.0476	5	7	0.0440
	**11000**	5	7	0.0770	6	8	0.0565	5	7	0.0469	6	8	0.0768	5	7	0.0447
	**13000**	5	7	0.0691	6	8	0.1026	5	7	0.0889	6	8	0.0688	5	7	0.0541

**P9**	**1000**	273	556	0.3340	428	922	0.3705	253	271	0.1782	240	259	0.3296	694	1014	0.9078
	**3000**	306	670	0.5481	484	1076	0.8123	345	367	0.5969	467	488	1.1298	456	653	0.8818
	**5000**	293	622	0.9203	646	1439	1.6881	313	332	0.7301	283	301	1.1130	819	1185	3.8548
	**7000**	355	758	1.5064	396	893	1.9556	408	425	1.4273	399	418	2.3700	628	925	4.1467
	**9000**	438	976	2.5387	430	933	2.7037	387	407	2.0961	323	341	2.7924	573	826	5.8853
	**11000**	467	1028	3.2065	391	838	2.9445	337	356	2.0743	340	360	3.3758	392	556	3.7520
	**13000**	384	832	2.8887	272	543	2.1416	310	325	2.0715	200	215	2.1939	513	745	6.9349

**Table 4:** Numerical results of ASSA algorithms and SSGM algorithms on large–scale problems 10 - 18 with their dimensions.

PROBLEMS	DIM	ASSA1			SSGM1			ASSA2			SSGM2			ASSA3		
		ITER	FEVAL	CPU–TIME	ITER	FEVAL	CPU–TIME	ITER	FEVAL	CPU–TIME	ITER	FEVAL	CPU–TIME	ITER	FEVAL	CPU–TIME
**P10**	**1000**	4	5	0.0864	4	5	0.0100	4	5	0.0148	4	5	0.0150	4	5	0.0203
	**3000**	4	5	0.0144	4	5	0.0382	4	5	0.0091	4	5	0.0269	4	5	0.0186
	**5000**	4	5	0.0441	4	5	0.0510	4	5	0.0194	4	5	0.0326	4	5	0.0242
	**7000**	4	5	0.0341	4	5	0.0354	4	5	0.0291	4	5	0.0519	4	5	0.1436
	**9000**	4	5	0.0558	4	5	0.0345	4	5	0.0474	4	5	0.0716	4	5	0.1129
	**11000**	4	5	0.0585	4	5	0.0904	4	5	0.0821	4	5	0.0523	4	5	0.0546
	**13000**	4	5	0.0746	4	5	0.0644	4	5	0.1171	4	5	0.0854	4	5	0.0774
**P11**	**1000**	4	5	0.0364	4	5	0.0031	4	5	0.0081	4	5	0.0041	4	5	0.0209
	**3000**	4	5	0.0096	4	5	0.0102	4	5	0.0214	4	5	0.0114	4	5	0.0256
	**5000**	4	5	0.0291	4	5	0.0166	4	5	0.0147	4	5	0.0138	4	5	0.0168
	**7000**	4	5	0.0180	4	5	0.0163	4	5	0.0235	4	5	0.0184	4	5	0.0475
	**9000**	4	5	0.0588	4	5	0.0258	4	5	0.0417	4	5	0.0289	4	5	0.0608
	**11000**	4	5	0.0240	4	5	0.0300	4	5	0.0347	4	5	0.0492	4	5	0.0464
	**13000**	4	5	0.0341	4	5	0.0480	4	5	0.0648	4	5	0.0474	4	5	0.0687
**P12**	**1000**	2	3	0.0685	2	3	0.0051	2	3	0.0079	2	3	0.0089	2	3	0.0100
	**3000**	1	2	0.0061	1	2	0.0048	1	2	0.0036	1	2	0.0043	1	2	0.0136
	**5000**	1	2	0.0099	1	2	0.0085	1	2	0.0048	1	2	0.0123	1	2	0.0067
	**7000**	1	2	0.0163	1	2	0.0065	1	2	0.0049	1	2	0.0066	1	2	0.0082
	**9000**	1	2	0.0103	1	2	0.0114	1	2	0.0184	1	2	0.0123	1	2	0.0097
**P13**	**1000**	26	52	0.1101	26	52	0.0290	29	45	0.0305	29	45	0.0503	31	54	0.0477
	**3000**	26	55	0.0834	26	55	0.1085	29	48	0.0479	29	48	0.1119	26	50	0.0495
	**5000**	30	62	0.1575	30	62	0.1749	28	49	0.1847	28	49	0.1152	27	51	0.1838
	**7000**	25	56	0.2409	25	56	0.1829	29	50	0.2571	29	50	0.1678	32	59	0.1780
	**9000**	29	63	0.2558	29	63	0.2257	28	53	0.2789	28	53	0.2955	27	53	0.2893
	**11000**	25	59	0.1936	25	59	0.2356	30	52	0.3664	30	52	0.3051	31	61	0.4500
	**13000**	30	65	0.2439	30	65	0.4019	29	52	0.2868	29	52	0.4385	31	59	0.4889
**P14**	**1000**	4	6	0.0533	5	7	0.0039	4	6	0.0037	5	7	0.0140	4	6	0.0043
	**3000**	4	6	0.0175	5	7	0.0116	4	6	0.0131	5	7	0.0214	4	6	0.0280
	**5000**	4	6	0.0183	5	7	0.0315	4	6	0.0182	5	7	0.0236	4	6	0.0157
	**7000**	4	6	0.0152	5	7	0.0273	4	6	0.0307	5	7	0.0293	4	6	0.0560
	**9000**	4	6	0.0242	5	7	0.0365	4	6	0.0292	5	7	0.1082	4	6	0.0267
	**11000**	4	6	0.0696	5	7	0.0696	4	6	0.0327	5	7	0.0334	4	6	0.0298
	**13000**	4	6	0.0449	5	7	0.0743	4	6	0.0709	5	7	0.0394	4	6	0.0340
**P15**	**1000**	26	33	0.2736	76	122	0.2511	36	43	0.1526	57	71	0.2341	31	38	0.1591
	**3000**	24	31	0.1887	F	F	F	36	43	0.1628	44	57	0.6031	34	41	0.2349
	**5000**	25	32	0.5459	F	F	F	34	41	0.3250	45	58	0.6044	34	41	0.5982
	**7000**	25	32	0.3477	F	F	F	37	44	0.5041	48	61	0.6758	32	39	0.7530
	**9000**	27	34	0.4433	F	F	F	37	44	0.4586	45	58	0.9181	35	42	0.7845
	**11000**	26	33	0.5158	F	F	F	35	42	0.6068	47	60	1.0753	34	41	1.1040
	**13000**	29	36	0.5643	F	F	F	35	42	0.6318	47	60	1.2024	34	41	1.0577
**P16**	**1000**	16	29	0.1196	16	30	0.0104	16	25	0.0321	17	28	0.0216	13	22	0.0171
	**3000**	16	29	0.0520	16	30	0.0584	16	25	0.0458	17	28	0.0358	13	22	0.0312
	**5000**	16	29	0.0654	16	30	0.0838	16	25	0.0928	17	28	0.1675	13	22	0.0831
	**7000**	16	29	0.1123	16	30	0.0834	16	25	0.0954	17	28	0.0990	13	22	0.1071
	**9000**	16	29	0.1325	16	30	0.1218	16	25	0.1648	17	28	0.0994	13	22	0.1498
	**11000**	16	29	0.1117	16	30	0.1210	16	25	0.1841	17	28	0.1260	13	22	0.1710
	**13000**	16	29	0.1850	16	30	0.2385	16	25	0.2639	17	28	0.1582	13	22	0.3017
**P17**	**1000**	2	9	0.1048	2	9	0.0046	2	9	0.0104	2	9	0.0091	2	9	0.0223
	**3000**	2	9	0.0065	2	9	0.0065	2	9	0.0080	2	9	0.0108	2	9	0.0099
	**5000**	2	9	0.0689	2	9	0.0093	2	9	0.0144	2	9	0.0205	2	9	0.0158
	**7000**	2	9	0.0228	2	9	0.0283	2	9	0.0310	2	9	0.0531	2	9	0.0418
	**9000**	2	9	0.0187	2	9	0.0237	2	9	0.0206	2	9	0.0347	2	9	0.0360
	**11000**	2	9	0.0210	2	9	0.0622	2	9	0.0340	2	9	0.0382	2	9	0.0723
	**13000**	2	9	0.0387	2	9	0.0332	2	9	0.0495	2	9	0.0564	2	9	0.0390
**P18**	**1000**	24	34	0.1386	F	F	F	F	F	F	22	29	0.0615	21	28	0.0992
	**3000**	24	34	0.1585	F	F	F	F	F	F	22	29	0.1494	21	28	0.1959
	**5000**	24	34	0.1509	F	F	F	F	F	F	22	29	0.2431	21	28	0.1483
	**7000**	24	34	0.3281	29	43	0.1560	21	28	0.1371	22	29	0.3078	21	28	0.4024
	**9000**	24	34	0.3605	F	F	F	14	21	0.1691	22	29	0.3780	21	28	0.4940
	**11000**	24	34	0.4601	F	F	F	14	21	0.1947	22	29	0.2749	21	28	0.4075
	**13000**	24	34	0.3309	F	F	F	14	21	0.2054	22	29	0.3192	21	28	0.3085

**Table 5:** Numerical results of ASSA algorithms and SSGM algorithms on large–scale problems 19 - 27 with their dimensions.

PROBLEMS	DIM	ASSA1			SSGM1			ASSA2			SSGM2			ASSA3		
		ITER	FEVAL	CPU–TIME	ITER	FEVAL	CPU–TIME	ITER	FEVAL	CPU–TIME	ITER	FEVAL	CPU–TIME	ITER	FEVAL	CPU–TIME
**P19**	**1000**	124	227	0.2454	99	175	0.0548	F	F	F	61	68	0.0516	75	102	0.0666
	**3000**	72	116	0.1881	440	863	0.5243	70	78	0.0692	63	85	0.0733	67	83	0.1909
	**5000**	75	107	0.2482	121	217	0.3774	F	F	F	64	72	0.1592	83	111	0.3084
	**7000**	69	106	0.3913	262	503	1.2322	68	79	0.2577	72	82	0.1816	111	149	0.5482
	**9000**	54	84	0.4995	293	595	1.7250	75	86	0.5050	62	73	0.3605	116	160	1.1176
	**11000**	143	265	1.7144	157	271	1.0503	69	81	0.5512	73	83	0.4355	80	104	0.7283
	**13000**	113	206	1.5045	131	251	0.9464	77	89	0.5620	59	66	0.3730	54	64	0.4717
**P20**	**1000**	1	2	0.0737	1	2	0.0013	1	2	0.0035	1	2	0.0057	1	2	0.0071
	**3000**	1	2	0.0101	1	2	0.0054	1	2	0.0043	1	2	0.0027	1	2	0.0024
	**5000**	1	2	0.0119	1	2	0.0063	1	2	0.0083	1	2	0.0040	1	2	0.0103
	**7000**	1	2	0.0056	1	2	0.0058	1	2	0.0053	1	2	0.0043	1	2	0.0063
	**9000**	1	2	0.0158	1	2	0.0067	1	2	0.0108	1	2	0.0065	1	2	0.0087
	**11000**	1	2	0.0096	1	2	0.0224	1	2	0.0098	1	2	0.0105	1	2	0.0109
	**13000**	1	2	0.0135	1	2	0.0085	1	2	0.0127	1	2	0.0054	1	2	0.0047
**P21**	**1000**	13	18	0.1035	13	17	0.0345	14	18	0.0222	11	15	0.0217	10	14	0.0113
	**3000**	13	18	0.0181	13	17	0.0294	14	18	0.0220	11	15	0.0270	10	14	0.0276
	**5000**	13	18	0.0522	13	17	0.0407	14	18	0.0427	11	15	0.0427	10	14	0.0549
	**7000**	13	18	0.1027	13	17	0.0551	14	18	0.0672	11	15	0.0365	10	14	0.0926
	**9000**	13	18	0.1262	13	17	0.0888	14	18	0.1334	11	15	0.0618	10	14	0.0540
	**11000**	13	18	0.1401	13	17	0.0895	14	18	0.0806	11	15	0.1284	10	14	0.1137
	**13000**	13	18	0.1009	13	17	0.0926	14	18	0.1125	11	15	0.0749	10	14	0.1147
**P22**	**1000**	17	32	0.1077	22	49	0.0174	17	32	0.0331	21	36	0.0307	17	32	0.0148
	**3000**	17	32	0.0273	22	49	0.0501	17	32	0.0399	21	36	0.0356	17	32	0.0901
	**5000**	17	32	0.0324	22	49	0.1321	17	32	0.0758	21	36	0.1068	17	32	0.0893
	**7000**	17	32	0.0856	22	49	0.1598	17	32	0.1456	21	36	0.1223	17	32	0.1837
	**9000**	17	32	0.1175	22	49	0.1947	17	32	0.2250	21	36	0.2084	17	32	0.2152
	**11000**	17	32	0.1803	22	49	0.2920	17	32	0.1969	21	36	0.2336	17	32	0.1968
	**13000**	17	32	0.2482	22	49	0.3538	17	32	0.2299	21	36	0.1970	17	32	0.2889
**P23**	**1000**	4	6	0.0419	5	7	0.0049	4	6	0.0158	5	7	0.0082	4	6	0.0145
	**3000**	4	6	0.0138	5	7	0.0196	4	6	0.0211	5	7	0.0214	4	6	0.0262
	**5000**	4	6	0.0143	5	7	0.0244	4	6	0.0205	5	7	0.0234	4	6	0.0450
	**7000**	4	6	0.0304	5	7	0.0717	4	6	0.0285	5	7	0.0443	4	6	0.0317
	**9000**	4	6	0.0273	5	7	0.0443	4	6	0.1002	5	7	0.0402	4	6	0.0491
	**11000**	4	6	0.0221	5	7	0.0552	4	6	0.0460	5	7	0.0998	4	6	0.0366
	**13000**	4	6	0.0462	5	7	0.0557	4	6	0.0362	5	7	0.0710	4	6	0.0721
**P24**	**1000**	21	35	0.1239	15	29	0.0076	21	35	0.0273	15	29	0.0168	21	35	0.0394
	**3000**	16	31	0.0655	22	47	0.0445	16	31	0.0445	19	34	0.0348	16	31	0.0576
	**5000**	17	32	0.0969	22	48	0.0715	17	32	0.0500	20	35	0.0735	17	32	0.0902
	**7000**	17	32	0.1311	23	49	0.1438	17	32	0.0811	20	35	0.0653	17	32	0.0985
	**9000**	17	32	0.2278	23	49	0.1736	17	32	0.1244	20	35	0.2216	17	32	0.2913
	**11000**	17	32	0.2305	20	47	0.2080	17	32	0.1851	20	35	0.2208	17	32	0.2307
	**13000**	17	32	0.1758	20	47	0.3059	17	32	0.2854	21	36	0.2856	17	32	0.2278
**P25**	**1000**	17	29	0.0882	21	34	0.0628	18	30	0.0201	18	30	0.0343	17	29	0.0494
	**3000**	20	35	0.0741	26	41	0.1577	21	36	0.0540	21	36	0.0474	20	35	0.1115
	**5000**	17	29	0.1038	19	31	0.1272	17	29	0.1251	19	31	0.1644	17	29	0.1261
	**7000**	18	31	0.1181	20	33	0.2038	18	31	0.1356	20	33	0.1977	18	31	0.1256
	**9000**	19	33	0.1547	20	34	0.1436	19	33	0.2348	21	35	0.1163	19	33	0.2147
	**11000**	19	33	0.2715	20	34	0.2641	20	34	0.1575	22	36	0.1926	20	34	0.2546
	**13000**	19	34	0.2095	21	36	0.2056	20	35	0.3355	22	37	0.1945	20	35	0.2838
**P26**	**1000**	336	735	0.6566	314	703	0.3193	272	291	0.1699	230	248	0.1212	441	636	0.6060
	**3000**	316	666	1.1565	283	585	0.5389	248	275	0.4805	199	218	0.2794	393	564	1.1398
	**5000**	302	647	1.5136	293	601	1.1529	260	282	0.7733	312	327	0.7131	468	654	2.2223
	**7000**	468	984	3.8041	387	833	1.5875	266	291	1.0078	264	280	1.4697	437	631	1.6654
	**9000**	339	714	3.2110	318	690	1.6699	188	212	0.8802	280	300	1.2895	542	781	2.8109
	**11000**	270	554	3.0366	263	537	1.7500	347	360	2.0573	297	312	1.3763	503	685	3.1762
	**13000**	254	512	1.7371	472	981	3.4959	293	314	1.7208	277	294	1.5208	401	565	4.1690
**P27**	**1000**	2	23	0.0913	2	23	0.0089	2	23	0.0138	2	23	0.0116	2	23	0.0249
	**3000**	2	27	0.0230	2	27	0.0396	2	27	0.0237	2	27	0.0177	2	27	0.0188
	**5000**	2	28	0.0542	2	28	0.0621	2	28	0.0460	2	28	0.0502	2	28	0.0496
	**7000**	2	29	0.0684	2	29	0.0641	2	29	0.0913	2	29	0.0489	2	29	0.0550
	**9000**	2	30	0.1293	2	30	0.0781	2	30	0.0717	2	30	0.0733	2	30	0.0986
	**11000**	2	30	0.1330	2	30	0.1141	2	30	0.1279	2	30	0.1292	2	30	0.1260
	**13000**	2	31	0.1211	2	31	0.1041	2	31	0.1184	2	31	0.0579	2	31	0.1242

**Table 6:** Numerical results of ASSA algorithms and SSGM algorithms on small scale problems 28 - 35 with their dimensions.

PROBLEMS	DIM	ASSA1			SSGM1			ASSA2			SSGM2			ASSA3		
		ITER	FEVAL	CPU–TIME	ITER	FEVAL	CPU–TIME	ITER	FEVAL	CPU–TIME	ITER	FEVAL	CPU–TIME	ITER	FEVAL	CPU–TIME
**P28**	n=2, m=3	F	F	F	F	F	F	9	27	0.0123	F	F	F	9	48	0.0113
**P29**	n=2, m=20	F	11	0.0248	1	7	0.0116	1	11	0.0078	1	7	0.0128	1	11	0.0051
**P30**	n=3, m=10	35	56	0.0298	F	F	F	17	22	0.0193	39	52	0.0341	13	18	0.0180
**P31**	n=2, m=3	8	11	0.0485	10	13	0.0048	10	13	0.0048	8	11	0.0213	8	11	0.0051
**P32**	n=2, m=2	F	F	F	49	127	0.0310	F	F	F	70	172	0.0331	72	125	0.0110
**P33**	n=2, m=3	11	27	0.0184	8	24	0.0151	7	23	0.0216	9	25	0.0061	14	33	0.0067
**P34**	n=2, m=2	26	61	0.0166	57	145	0.0196	23	38	0.0104	25	45	0.0109	38	70	0.0205
**P35**	n=2, m=3	25	42	0.0376	28	47	0.0140	26	32	0.0042	29	43	0.0133	25	38	0.0106

It can be seen from the data reported in Tables 3, 4, 5 and 6, that ASSA3, i.e. Algorithm 1 with the choice $\alpha_{k}^{M3}$, solved all the considered test problems successfully. However, each of the remaining algorithms recorded failure in at least 3 instances. In comparison with ASSA1 and ASSA2 algorithms, the algorithms SSGM1 and SSGM2 of [20] have had the highest number of failures in the course of solving the test problems. That means fusing a better approximation of the Hessian defined in equation (2.8) into the BB parameters coupled with the safeguarding technique resulted in an improved numerical performance to some extent.

The reported results in Tables 3, 4, 5 and 6 are summarized with the aid of well-known Dolan and Moré [28] performance profile. The comparison is conducted based on the following three metrics: *ITER, FVAL* and *CPU–TIME.*

**Figure 1:**
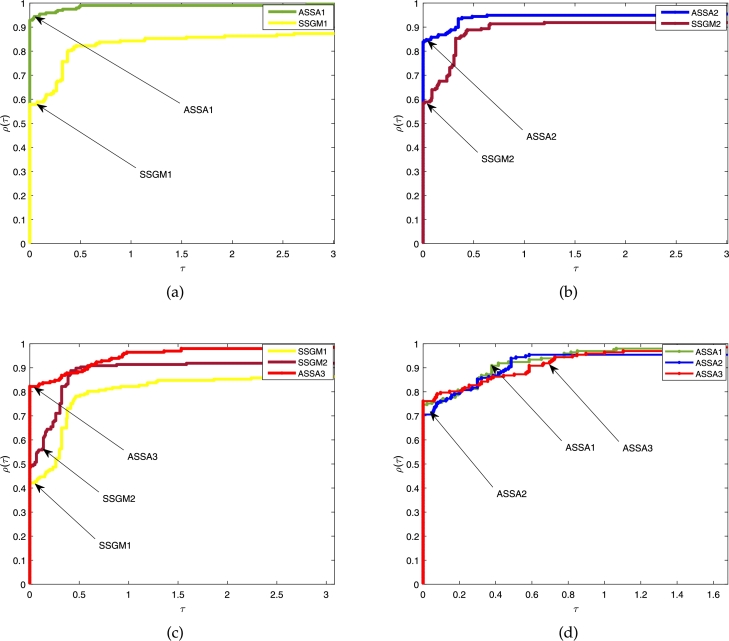
The above Fig. show comparisons based on the *ITER.* In 1(a) the results of ASSA1 and SSGM1 are compared. Similarly, ASSA2 and SSGM2 are also compared in 1(b). Moreover, in 1(c), ASSA3 is compared with SSGM1 and SSGM2, and all the three proposed algorithms, ASSA1, ASSA2 and ASSA3 are compared in 1(d).

**Figure 2:**
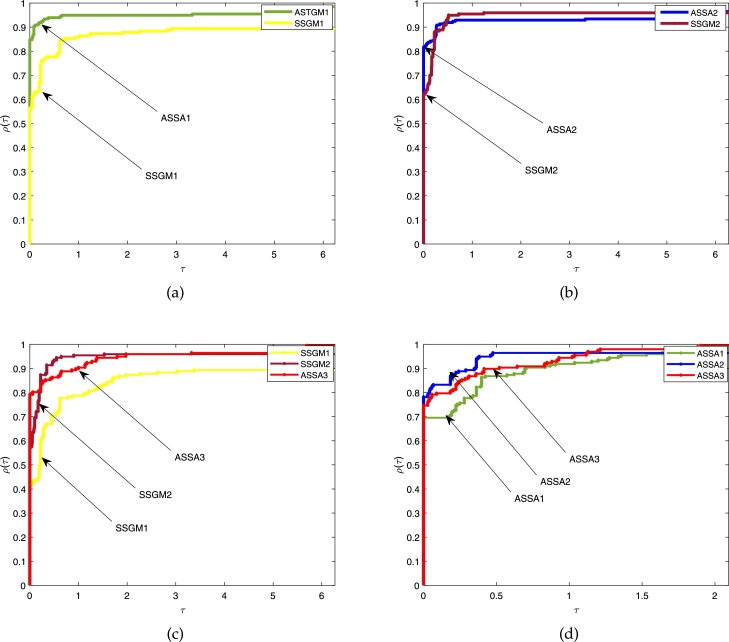
Furthermore, the above Fig. show comparisons based on the *FVAL.* In 2(a) the results of ASSA1 and SSGM1 are compared. Similarly, ASSA2 and SSGM2 are also compared in 2(b). Moreover, in 2(c), ASSA3 is compared with SSGM1 and SSGM2, and all the three proposed algorithms, ASSA1, ASSA2 and ASSA3 are compared in 2(d).

**Figure 3:**
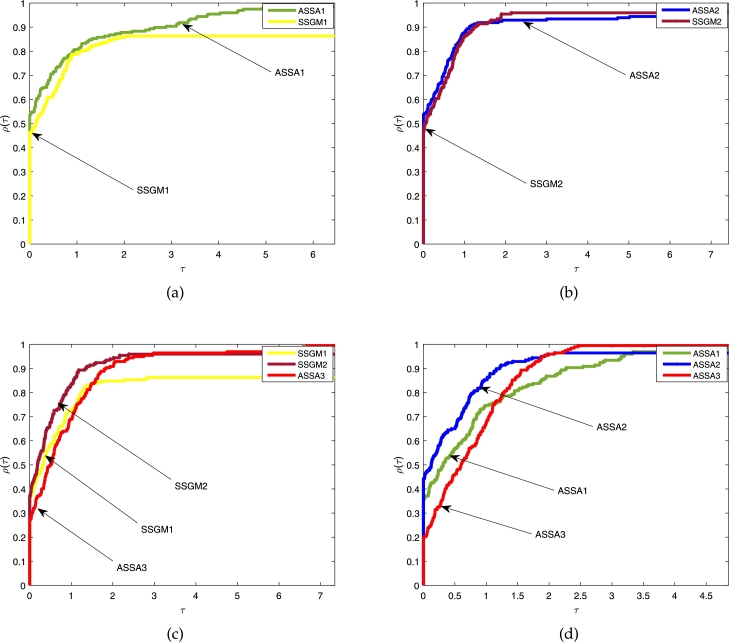
More so, the above Fig. show comparisons based on the *CPU-TIME*. In 3(a) the results of ASSA1 and SSGM1 are compared. Similarly, ASSA2 and SSGM2 are also compared in 3(b). Moreover, in 3(c), ASSA3 is compared with SSGM1 and SSGM2, and all the three proposed algorithms, ASSA1, ASSA2 and ASSA3 are compared in 3(d).

Figs. 1a, 2a and 3a show the comparison between the proposed ASSA1 and SSGM1; Figs. 1b, 2b and 3b are the comparison between proposed ASSA2 and SSGM2; Figs. 1c, 2c and 3c are the comparison between the proposed ASSA3 and SSGM1; SSGM2 and Figs. 1d, 2d and 3d are the comparison among the three proposed algorithms ASSA1, ASSA2 and ASSA3.

In terms of *ITER*, Fig. 1a–1c, show that the curves formed by our algorithms ASSA1, ASSA2, and ASSA3 stay longer on the *vertical axis* with a success rate of about 93%, 84%, and 82% respectively, as displayed by the height of their performance profile for τ>0.6. These show that our algorithms outperform their main competitors, SSGM1 and SSGM2. While Fig. 1d shows that our three proposed algorithms are competitive where ASSA3 performs slightly better than ASSA1 and ASSA2. Therefore, the ASSA3 algorithm can be considered the most *reliable* of all the algorithms since it was able to solve all the problems under consideration.

Moreover, with regards to *FVAL*, we can see in Fig. 2a–2c that ASSA1, ASSA2, and ASSA3 algorithms won about 85%, 83%, and 79%, respectively of the experiments in comparison to SSGM1 and SSGM2 algorithms. On the other hand, a comparison between the three proposed algorithms, reported in Fig. 2d shows that ASSA2 performs moderately better than its competitors ASSA1 and ASSA3.

Furthermore, regarding the CPU-TIME, Fig. 3a–3b show that the proposed algorithms ASSA1 and ASSA2 are very competitive against SSGM1 and SSGM2, where ASSA1 and ASSA2 perform slightly better. Hence, our algorithms are more *efficient* than their respective counterparts. Although ASSA3 achieves better results than ASSA1, ASSA2, SSGM1, and SSGM2 in terms of *ITER* and solved all the problems under consideration, it, however, loss to them based on *CPU–TIME.* Considering everything, we can see that the proposed algorithms have a better computational performance, and in particular, ASSA3 solved all the problems in all the instances.

## 5 Conclusions

We have proposed three structured spectral gradient algorithms for solving nonlinear least-squares problems. We built the three algorithms by incorporating the structured vector $\gamma_{k-1}$ into three different spectral parameters. The structured vector, $\gamma_{k-1}$ is obtained by approximating the Hessian of the objective function such that the secant equation is satisfied. We implemented three algorithms without the need to create or store matrices throughout the iteration process. That makes them suitable for large–scale problems. Moreover, we came up with a safeguarding strategy, different from that of [20], to avoid negative curvature directions. Numerical experiments presented in Section 4 have shown that our proposed algorithms work well and perform better than SSGM1 and SSGM2 of [20]. Furthermore, the implementation of Algorithm 1 using $\alpha_{k}^{M3}$, that is, the geometric mean of $\alpha_{k}^{M1}$ and $\alpha_{k}^{M2}$, proved to be more efficient as it successfully solved all the test problems considered in our numerical experiments without any failure. Our future study will include using a two-step approach of [29] to solve non-linear least-squares problems and applying these algorithms to motion control problems [30], [31], [32].

## Declarations

### Author contribution statement

M.M. Yahaya: Conceived and designed the experiments; Performed the experiments; Wrote the paper.

P. Kumam: Analyzed and interpreted the data; Wrote the paper.

A.M. Awwal: Conceived and designed the experiments; Analyzed and interpreted the data; Wrote the paper.

S. Aji: Performed the experiments; Wrote the paper.

### Funding statement

The authors acknowledge the financial support provided by the Petchra Pra Jom Klao Scholarship of 10.13039/501100004705King Mongkut's University of Technology Thonburi (KMUTT) and Center of Excellence in Theoretical and Computational Science (TaCSCoE), KMUTT (TaCS-CoE 2021). The first author got support from Petchra Pra Jom Klao Masters Research Scholarship from 10.13039/501100004705King Mongkut's University of Technology Thonburi (Contract No. 5/2562). Also, Aliyu Muhammed Awwal would like to thank the Postdoctoral Fellowship from King Mongkut's University of Technology Thonburi (KMUTT), Thailand. Moreover, this research project is supported by 10.13039/501100017170Thailand Science Research and Innovation (TSRI) Basic Research Fund: Fiscal year 2021 under project number 64A306000005.

### Data availability statement

Data included in article/supplementary material/referenced in article.

### Declaration of interests statement

The authors declare no conflict of interest.

### Additional information

No additional information is available for this paper.
